# Supplementary material for: Murine T-cell receptor OT-I exhibits imperfect discrimination between foreign and self-antigens
Source: EMBO J. 2025 Nov 26;45(2):394–416. doi: 10.1038/s44318-025-00644-5 (PMC12811252; doi:10.1038/s44318-025-00644-5)
Supplement: Supplementary file 1 — Appendix [file 44318_2025_644_MOESM1_ESM.pdf]

Appendix for:

Murine T-cell receptor OT-I exhibits imperfect discrimination between foreign and self-antigens

Table of Contents

Appendix Figure S1 - Page 2
Appendix Figure S2 - Page 3

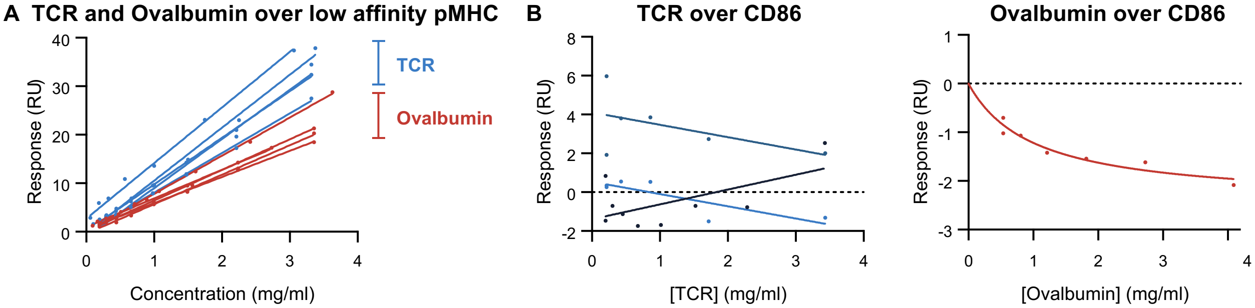

Appendix Figure S1: An irrelevant protein (ovalbumin) can bind heterotrimeric pMHC but not monomeric CD86. (A) Steady-state binding of ovalbumin or OT-I analytes over pMHC surfaces. Note that the TCR displays higher binding than ovalbumin suggesting that it binds both inactive and active pMHC whereas ovalbumin binds only inactive pMHC. (B) Steady-state binding of TCR (left) or ovalbumin (right) over CD86 surfaces reveals no detectable binding. Negative values can arise from modest refractive index effects. Note scale differences compared to panel A.

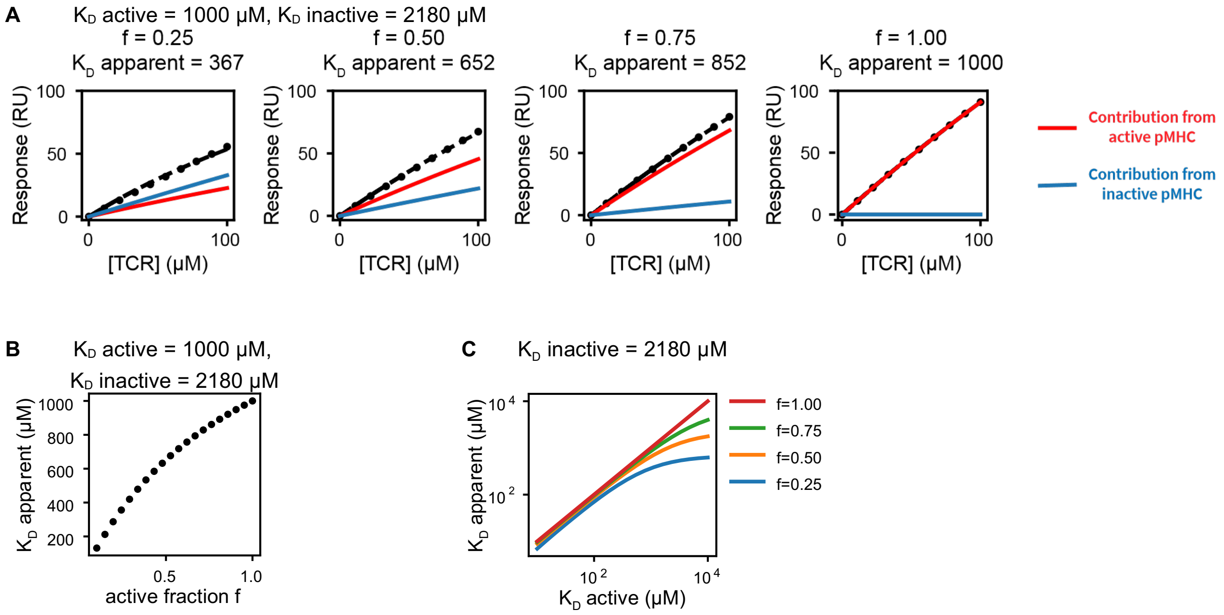

Appendix Figure S2: Simulations highlight the impact of the fraction of active pMHC (f) on apparent K_D values. (A) Simulated TCR binding curves to surfaces containing variable amounts of active and inactive pMHCs (columns). The overall binding signal (black) was fit to a 1:1 binding model to estimate the apparent K_D. (B) Apparent K_D values derived from fitting the simulated curves in (A) plotted against the fraction of active pMHC (f). The apparent K_D diverges from the active K_D as f decreases. (C) Apparent K_D values plotted against the true active K_D for different f, demonstrating the dependency of apparent K_D on both f and the active K_D.
