# Supplementary material for: Murine T-cell receptor OT-I exhibits imperfect discrimination between foreign and self-antigens
Source: EMBO J. 2025 Nov 26;45(2):394–416. doi: 10.1038/s44318-025-00644-5 (PMC12811252; doi:10.1038/s44318-025-00644-5)
Supplement: Supplementary file 9 — Expanded View Figures [file 44318_2025_644_MOESM9_ESM.pdf]

Expanded View Figures

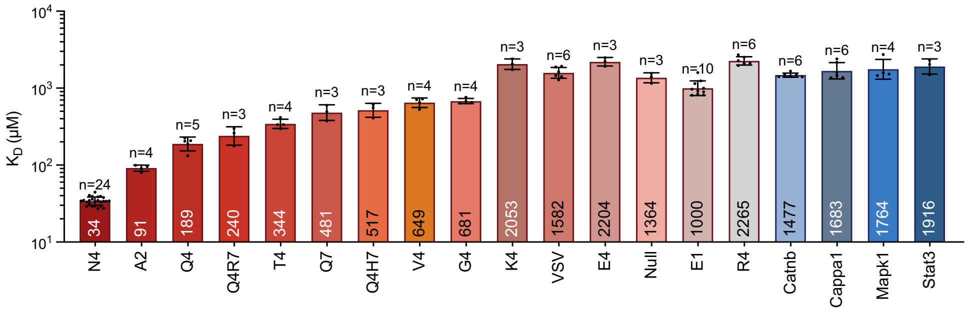

Figure EV1. Apparent K_D values for OT-I specific peptides.

The K_D values were obtained by fitting the steady-state binding using a 1:1 model with B_{max} constrained to value obtained from the B2M or Y3 antibody binding using the standard curve (see Fig. 1 and "Methods"). Geometric mean is displayed within the bars, mean values, error and *N* are listed in Table 1.

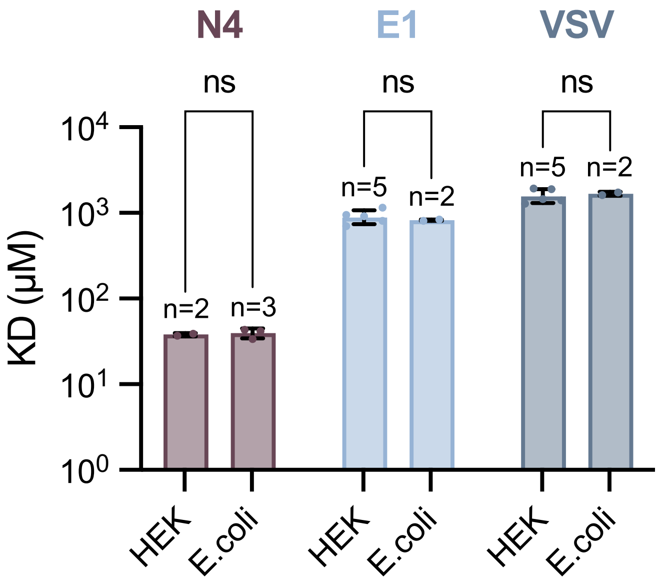

Figure EV2. The OT-I TCR displays similar affinity to pMHC produced in *E. coli* and HEK293T cells.

The *E. coli*-produced pMHC were produced in-house, whereas the HEK293T-produced pMHC were supplied by the NIH tetramer facility. Bars show mean values ± SD. Statistical significance was determined using a two-way ANOVA test.

 © The Author(s)

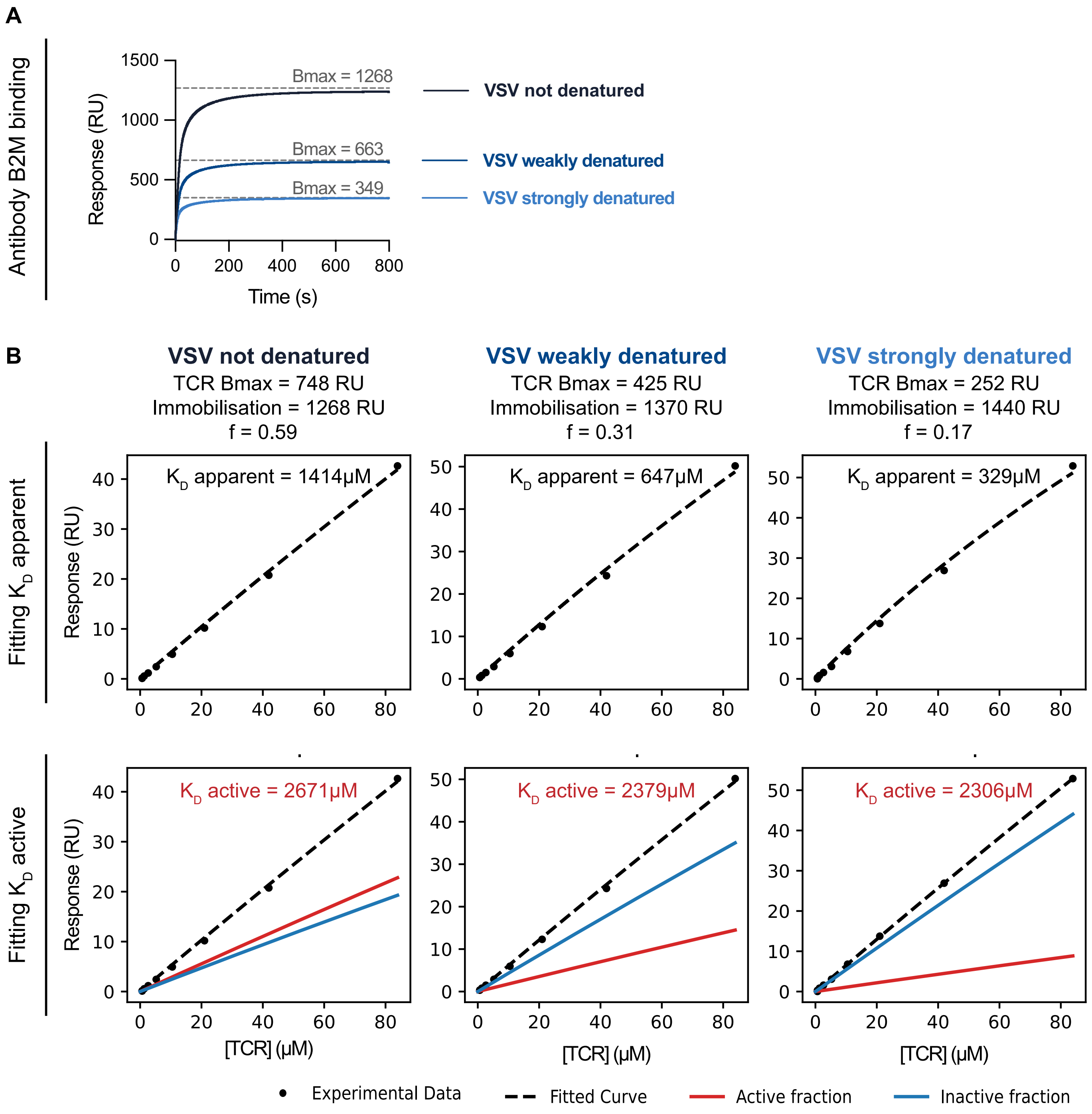

Figure EV3. Calculating the active K_D provides similar results for OT-I binding to surfaces with large differences in the fraction of active pMHC.

The VSV pMHC was immobilised at similar levels on 3 flow cells in SPR before inducing denaturing by a short (weakly denatured) or long (strongly denatured) injection of glycine solution (pH 1.7). (**A**) B2M antibody binding curves to the three VSV pMHC surfaces. The TCR B_{max} was estimated using the standard curve in Fig. 1B. (**B**) Steady-state TCR binding response to the 3 surfaces (columns). To determine the apparent K_D, the data was fit with a 1:1 binding model with constrained B_{max} to determine K_D apparent (top row). To determine the active K_D, the workflow in Fig. 2G–I was used, where the fraction of active pMHC was calculated from the ratio of TCR B_{max} to pMHC immobilisation, and K_D inactive was fixed to 2180 µM. While the apparent K_D displayed large differences, the active K_D produced consistent results across all VSV pMHC surfaces.

© The Author(s)

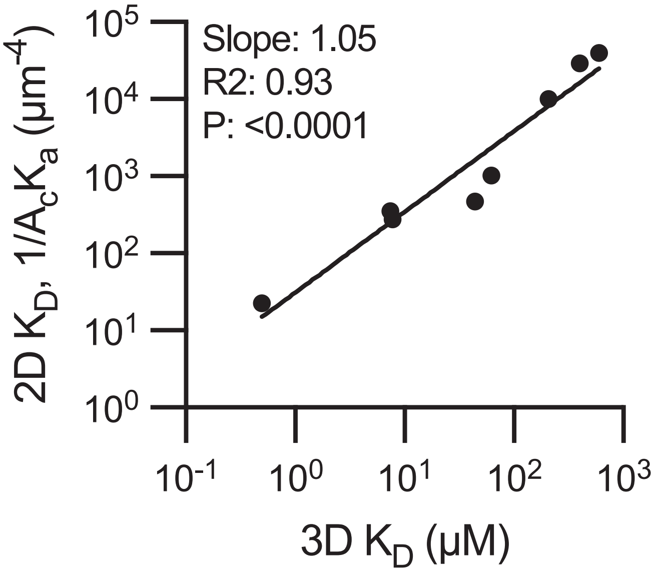

Figure EV4. Quantitative comparison of 2D and 3D affinities for the 1E6 TCR.

The log-transformed data was fitted with a linear regression. An *F* test was used to determine a *P* value for the null hypothesis that the slope is equal to zero. All data were taken from Cole et al (Cole et al, 2016).

 © The Author(s)

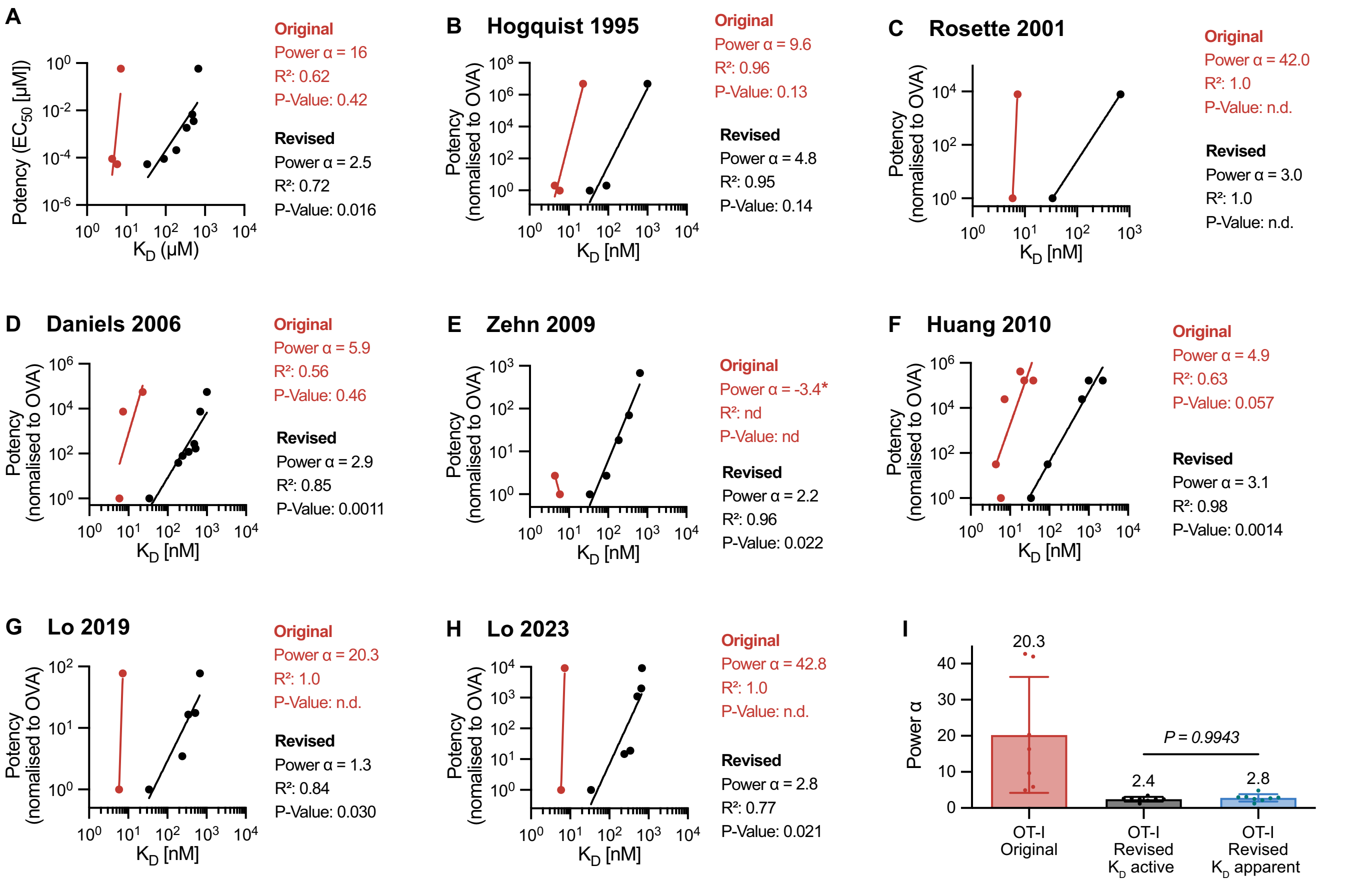

Figure EV5. Discriminatory power of OT-I TCR calculated with K_D apparent shows imperfect discrimination.

Plots show peptide potency over original or apparent K_D values. (**A**) Potency data from Fig. 5A. Data is mean of $N = 2$ independent experiments. (**B–H**) Published potency data from the indicated study over original or apparent K_D values. A power law (potency ~ $(K_D)^{\alpha}$) is fit to the data to estimate the discriminatory power α. A Pearson correlation is used to determine R^2 and P values on log-transformed values. (**I**) The discriminatory power from (**A–H**) in comparison with discriminatory power calculated with active K_D values. The P value is determined using a t-test. Number of data points included in each category: $N = 7$, $N = 8$, $N = 5$, $N = 15$ for OT-I Original, OT-I revised, other mouse TCRs and other human TCRs, respectively. Bars show mean values (also indicated above the bar) ± SD.

© The Author(s)
